# Bodies Coming Apart and Bodies Becoming Parts: Widening, Deepening, and Embodying Ontological (In)Security in the Context of the COVID-19 Pandemic

**DOI:** 10.1093/isagsq/ksab037

**Published:** 2021-11-09

**Authors:** Kandida Purnell

**Affiliations:** Richmond, The American International University in London, UK

## Abstract

This article widens and deepens the notion of ontological security and therefore both the scope of ontological security studies (OSS) within the discipline of international relations (IR) and ontological security theory (OST) writ large by introducing and explaining the implications of (re/dis)embodiment—the continually contested social–political process through which bodies come to be or not be and upon which everybody existentially and ontologically depends. Understood as both a source of and a threat to individual and collective bodies’ ontological security, in this article I explain how taking the process of (re/dis)embodiment into account entails widening and deepening OSS to allow for the consideration and appreciation of how individual and collective bodies are continually, simultaneously, materially, and ideationally contested. As a primarily theoretical contribution, this is done through an interdisciplinary approach bringing Achille Mbembe's necropolitical theory into conversation with Sara Ahmed's theses on willfulness and use and is illustrated through discussions on the body politics of the COVID-19 pandemic. In short, I argue that, under conditions of contemporary global necropolitics, individuals’ ontological security as bodies becomes increasingly threatened according to raced, classed, and gendered local–global hierarchies which determine the reduction and use of individuals to the status of parts within collectives that are themselves embodied and increasingly unfit for the purpose of healthy living, especially in a time of pandemic.

## Abstract (es)

Este artículo amplía y profundiza la noción de seguridad ontológica y, por tanto, el alcance de los estudios de seguridad ontológica (Ontological Security Studies, OSS) dentro de la disciplina de las Relaciones Internacionales (RI) y de la teoría de la seguridad ontológica (Ontological Security Theory, OST) en general, mediante la introducción y explicación de las implicaciones del (re/dis)embodiment o (re/des)encarnación (es decir, el proceso sociopolítico continuamente cuestionado a través del cual los cuerpos llegan a ser o no ser, y del cual todo cuerpo depende existencial y ontológicamente). En este artículo, explico cómo el tener en cuenta el proceso de (re/des)encarnación (entendido como una fuente y una amenaza para la seguridad ontológica de los cuerpos individuales y colectivos) implica ampliar y profundizar los OSS para permitir la consideración y la apreciación de cómo los cuerpos individuales y colectivos son continua y simultáneamente impugnados de forma material. Como contribución principalmente teórica, esto se hace a través de un enfoque interdisciplinario que pone en conversación la teoría necropolítica de Achille Mbembe con las tesis de Sara Ahmed sobre la voluntad y el uso, y se ilustra a través de discusiones sobre las políticas relacionadas con el cuerpo en el marco de la pandemia COVID-19. En resumen, sostengo que, bajo las condiciones de la necropolítica global contemporánea, la seguridad ontológica de los individuos como cuerpos se ve cada vez más amenazada en función de las jerarquías locales-globales de raza, clase y género que determinan la reducción y el uso de los individuos al estatus de partes dentro de colectivos, los cuales son en sí mismos encarnados y cada vez más inadecuados para el propósito de una vida saludable, especialmente en una época de pandemia.

## Abstract (fr)

Cet article élargit et approfondit la notion de sécurité ontologique, et donc à la fois l’étendue des études de la sécurité ontologique dans la discipline des relations internationales (RI) et l’étendue de la théorie de la sécurité ontologique au sens large. Pour ce faire, il introduit et explique les implications de la (ré/dés)incarnation, c'est-à-dire, le processus sociopolitique continuellement contesté par lequel les corps finissent par être ou ne pas être et dont chaque corps dépend existentiellement et ontologiquement. En la comprenant à la fois comme une source et une menace pour la sécurité ontologique des corps individuels et collectifs, j'explique comment la prise en compte du processus de (ré/dés)incarnation englobe un élargissement et un approfondissement des études de la sécurité ontologique pour permettre une prise en considération et une appréciation des manières dont les corps individuels et collectifs sont continuellement et simultanément contestés du point de vue matériel et idéationnel. Je me livre à cette contribution principalement théorique en adoptant une approche interdisciplinaire, qui établit un dialogue entre la théorie nécropolitique d'Achille Mbembe et les thèses de Sara Ahmed sur l'obstination et son usage, et je l'illustre par des discussions sur les politiques du corps qui sont intervenues durant la pandémie de COVID-19. En bref, je soutiens que, dans les conditions de la nécropolitique mondiale contemporaine, la sécurité ontologique des individus en tant que corps devient de plus en plus menacée par les hiérarchies locales et mondiales fondées sur la race, la classe et le genre, qui déterminent la réduction et l'utilisation des individus au statut de parties de collectifs qui sont eux-mêmes incarnés et de plus en plus impropres à l'objectif d'une vie saine, en particulier en période de pandémie.

## Introduction

In April 2021, the United Kingdom's Daily Mail Newspaper published claims that the Prime Minister (PM) Boris Johnson (cited in Walters 2021) had weighed in on policy options during the second wave of the pandemic exclaiming “no more fucking lockdowns—let the bodies pile high in their thousands.” The reported remark caused public outrage as Johnson depersonalized COVID-19 victims by referring to them as “just” bodies. However, it was not only the depersonalized reference to dead bodies that warrants Johnsons’ remarks’ inclusion here but also his willingness to let bodies *pile high* in their thousands, which I found telling and relevant toward explaining the breadth and depth of ontological insecurity that threatens embodied subjects not only at the surface level of identity and selfhood but also *all the way down* to the existential and fundamentally ontological level of being in itself. Indeed, the ontological (in)security outlined in this article takes into account what the COVID-19 pandemic is making clear—that embodied subjects live in a socially–politically constructed world where somebody seen and known as an absolutely loved and irreplaceable person to one can become reduced to and known as “only” their body. In short, in this article, I draw and build on interdisciplinary literature including the ever-increasing amount of international relations (IR) scholarship explicitly foregrounding bodies and embodiment (e.g., Marlin-Bennett, Marieke, and Jason 2010; Sylvester 2012; Auchter 2014; Fierke 2014; Wilcox 2015a; Fishel 2017; ; Baker 2020; Epstein 2021; Purnell 2021) as a means to “join up” ontological security studies (OSS) as it exists as an emerging, varied, and vibrant subdiscipline of IR with the notion of the (re/dis)embodiment.

As I explain in this article, truly *embodying* OSS entails (1) rethinking the body along interdisciplinary lines and (2) widening and deepening the notion of ontological (in)security to allow it to capture the non-agent centric process of the simultaneously material-ideational process of (re/dis)embodiment. As I then illustrate through this article's discussion of such wider and deeper ontological insecurities through the COVID-19 pandemic, by thoroughly embodying OSS as such it becomes possible to explain how material bodies—as well as *identity* and *selfhood—*are intensely politically contested rather than remaining “outside of” or “before” political acts. Indeed, the interdisciplinary notion of (re/dis)embodiment described in this article should, therefore, be considered as both a *source of* and a *threat to* actors’ ontological (in)security and as a theoretical tool that takes into account and allows for the scrutiny of contested, intensely political processes through which individual and collective bodies continually come to be, not be, or become other. Crucially, the embodied notion of ontological (in)security presented in this article contributes to OSS as well as ontological security theory (OST) writ large and namely the sociological–psychoanalytical accounts of ontological security made by Anthony Giddens (1991) and R.D. Laing (1990) upon which OSS in IR is inspired. This wider and deeper notion of ontological (in)security arises by making OST capable of accounting for the *simultaneously* discursive and materializing threats to individual and collective bodies’ identities and material being in the world.

Toward further explaining what is at stake in the process of (re/dis)embodiment and how the notion of ontological (in)security put forward in this article widens and deepens the existing OSS, in this article I draw on the contemporary necropolitical theory of Achille Mbembe (2003, 2019, 2020a, 2020b) and Sara Ahmed's work thesis on *Use* (2019) to argue that a broader and deeper notion of ontological (in)security is required to account for and explain the existential and ontological security source and threat that (re/dis)embodiment both provides and poses to both individual embodied subjects and collective bodies in the contemporary era and within the COVID-19 pandemic in particular. However, before doing so and illustrating the argument via discussions around the ontological (in)security politics of the COVID-19 pandemic, it is vital to situate my contribution within the subfield of OSS and discipline of IR.

### Toward Embodying OSS in IR

The embodied notion of ontological (in)security put forward in this article builds on the existing OSS—and especially on Nina Krickel-Choi's recent intervention calling out OSS for until now theorizing a “disembodied self” (Krickel-Choi 2021, 3)—in two ways: (1) OSS literature is able to connect the ontological insecurities of states and individuals and recognize that insecurity at one level may engender insecurity for the other and OSS tends to be concerned with and tackles the ontological security of *either* states *or* individuals. However, my approach is able to capture and analyze both individual embodied subjects *and* collective bodies simultaneously though it focuses on neither actor but rather on the ongoing process of (re)embodiment. (2) By centralizing the process of (re/dis)embodiment, my framework moves beyond dualist thinking such as that reflected in OSS concerned with “security not of the body but of the self” (Mitzen 2006a, 344). Thus, where OSS isolates the selfhood of either the individual or the state as the referent object of and keeps this “level” of (in)security separate from the physical security of bodies and states the ontological (in)security I outline in this article threatens *embodied* subjects and collective bodies (*which are simultaneously body and self*) at the existential and indeed ontological level of material being in itself.

To provide an overview of the works inspiring and enabling the embodiment of OSS done through this article, IR as a discipline has recently begun to shine a light on bodies by centering them explicitly entangled in machineries of violent warfare and atrocity (see, e.g., Auchter 2014; Fierke 2014; Wilcox 2015a; Fishel 2017; Baker 2020; Epstein 2021; Narozhna 2021; Purnell 2021). In particular, Wilcox's intervention *Bodies of Violence* (Wilcox 2015a) has interrogated contemporary practices of violent warfare and security as a means to explicitly theorize the subject of violence as embodied, making the central argument that “the bodies that practices of violence take as their object are deeply political bodies, constituted in reference to historical political conditions while at the same time acting upon our world” (2015a, 3). Wilcox has done this through a focus on the violently extreme embodied practices of hunger striking (Wilcox 2012), suicide bombing (Wilcox 2013), and drone strikes (Wilcox 2015b, 2017). These works have shown a light on how particular and most often extremely placed and exposed bodies are violently exposed and damaged and in doing so offer up obvious examples of (re/dis)embodiments. However, in my different aim to re-think *every body* (see Purnell 2021), in this article I build on body-centric IR to purport that such extreme examples in fact illustrate how any and *every* body is held together and can therefore come apart via the ongoing process of (re/dis)embodiment, which makes for the ontological depth of insecurity I envisage.

Beyond recent, body-centric works in IR, OSS is rooted in the sociological work of Anthony Giddens (1991, 38–39) who uses the term “ontological security” to refer to:

a person's fundamental sense of safety in the world and includes a basic trust of other people. Obtaining such trust becomes necessary in order for a person to maintain a sense of psychological well-being and avoid existential anxiety.

Here, isolating the necessity of “basic trust” for sustaining persons’ ontological security (the sum of maintaining “a sense of psychological well-being” and avoiding “existential anxiety” as Giddens provides it), this article develops Giddens’ foundational contribution by outlining a deeper and more nuanced understanding of the role of social phenomena including the implications of embodied subjects requiring mutual “basic trust” to establish and maintain their ontological security. This article also fleshes out the notion of ontological security itself by (1) widening the concept to capture embodied subjects and collectives and (2) deepening the concept to account for the ontological excess of embodied subjects and other beings, who rely on others to be as such and be continuously (re/dis)embodied.

With Giddens’ conception of ontological security in turn influenced by the psychoanalytic theory of R.D. Laing (1990) and described succinctly by Ben Rosher (2020) as “concerned with the ways in which we know who we are and our place in our socio-material worlds,” the theory taken up and utilized in contemporary IR's burgeoning OSS literature primarily emphasizes and scrutinizes a notion of security as very much bound up with the individual's sense of selfhood that allows or prevents them from maintaining their psychological well-being and indeed behaving as coherent entities within a social setting. As Rosher goes on, “we perform ourselves in the world through embedded routines and discourses which serve to ‘bracket out’ the underlying fact that life is contingent and largely beyond our control in order that we are able to ‘go on’ with the everydayness of life” (Rosher 2020). As has recently been (re)discovered within OSS, both Giddens and Laing *do* within their accounts implicitly acknowledge embodiment—how bodies and selves are mutually constituted (See Gustafsson and Krickel-Choi 2020; Krickel-Choi 2021.) However, as Nina Krickel-Choi (2021, 1) has recently chronicled, the notion of embodiment has been almost completely glossed over by IR's OSS as the “discipline-specific incorporation [of OST] has had consequences.” Indeed, given the discipline's purview and historically acute disembodiment, IR theorists drawing on OST often have primarily used and “scaled up” Giddens’ and Laing's work to understand state behavior (see, e.g., Mitzen 2006a, 2006b, 2008; Steele 2008, 2019; Gustafsson 2014, 2015; Behravesh 2018; Greve 2018; Behravesh 2018; Capan and Zarakol 2019; Ejdus 2020) while others more latterly—and reflecting intradisciplinary preferences—take their referent object as the individual, using OSS to scrutinize the very particularly, yet problematically, conceived and assumed individuals’ behavior, interactions, and (in)security (see, e.g., Browning 2018a, 2018b, 2019; Flockhart 2016; Innes 2017; Homolar and Scholz 2019). However, within both the state and the individual focused veins of OSS, there are contributions that pave the way for my own.

First, relevant for the notion of ontological (in)security presented in this article, within the individual focused vein of OSS, there are contributions detailing how the ontological (in)security of individuals feeds into their increasing/decreasing physical (in)security (see, e.g., debate between Kinnvall 2004 and Innes 2017). In this literature, there is some debate about whether “hybrid identity” itself manifests as a form of ontological insecurity that then feeds into physical insecurity, with Kinnvall (2004) warning that it does and Innes (2017, 394) taking a more ambivalent position arguing that “a number of identities can be held simultaneously, both producing and resolving insecurity in social interactions as they intersect.” However, the referent object within these contributions remains the individual's *identity*—the increasing/decreasing ontological (in)security of which may then influence a corresponding increase/decrease in that individual's physical security.

Second, Narozhna (2020, 559) has outlined a “non-reductionist” framework “allowing us to elucidate the links between an individual, the society, and the state,” which crucially recognizes and problematizes the “anthropomorphisation of the state” (Narozhna 2020) due to how it “shifts the locus of consciousness and intentionality from individuals to the state” (Narozhna 2020, 569). However, noticing that states are spoken about, appear as, and are treated as human is not exactly the same as arguing that collective political units that I prefer to refer to as *bodies politic* are not only anthropomorphized but also deeply and profoundly embodied. From my perspective, the body politic is so much more than the state's machineries and representatives, meaning while terms are often interchanged, bodies politic are not to be confused with IR's “main actor” of the state, as bodies politic are composed of lively state- and non-state-affiliated body parts. However, within IR, the literal embodiment of the collectives populating the international system has not been appreciated or explored until now.

As Wendt (2004, 289) has explained, IR scholars often personify the state while “personhood is a useful fiction, analogy, metaphor, or shorthand for something else.” In their intervention, Narozhna (2020, 568) also recognizes that “interlocking relationships” make way for the becoming of both individuals and states. However these relationships are not fleshed out to provide a robust ontological (in)security theory detailing *how* the process of (re/dis)embodiment brings both individual and collective into and out of being but rather takes a “relational view of agents and structures” (Narozhna 2020, 569), which are assumed to remain secure in themselves at the deep ontological level. Moreover, understood as more deeply associated than units in “relations” with one another, the approach to ontological (in)security presented in this article rather describes both individual(ized) and collective(ized) bodies as coconstitutive of one another and involved, not in a “relational” manner but in an ongoing process of *correspondence*—(re/dis)embodiment.

Underlying my conceptual preference for “correspondence” over a relational approach and indeed informing the concept of (re/dis)embodiment, which in turn comes to inform my wider approach to rethinking bodies (Purnell 2021), is Tim Ingold ‘s work (Ingold 2007, 2015, 2016) wherein bodies and things are described as “bundles of thread” (Ingold 2007, 42) and accordingly as corresponding with one another in ways that the “blob-like” (Ingold 2016, 8) bodies and things in relationships with one another (such as the individuals and states described by orthodox IR) simply cannot. Brought into IR by Umut Ozguc (2020) to rethink borders, Ingold (2015) crucially explains that in *corresponding*, lines (and the bodies and things they make) intermingle—weaving together and clinging to one another—in a process that is never over and hence my emphasis on how (re/dis)embodiment is an *ongoing* process making embodied subjects fundamentally insecure at the level of being in itself.

Judith Butler's theory of performativity (“the reiterative power of discourse to produce the phenomenon that it regulates and constrains” [Butler 1993, 2]) further informs the depth of the notion of ontological (in)security outlined in this article by underlining how operations of power(/knowledge) get literally under the skin to flesh bodies out. Indeed, Butler explains how the process of (re/dis)embodiment involves the ongoing use of force as Butler describes bodies as “forcibly materialized through time” (Butler 1993, 1). Furthermore, toward explaining exactly how material bodies come to be, in *Excitable Speech* (Butler 1997, 159), Butler's description of how “words enter the limbs, craft the gesture, bend the spine” (Butler 1997, 159) brings to light how the process of (re/dis)embodiment involves the discursive being forcibly fleshed out as bodies become, are undone, or become other than bodies. In sum then, the deeper notion of ontological (in)security put forward in this article is marked out by its utterly antifoundational and indeed Butlerian (Butler 1993, 2) epistemology and ontology wherein “the fixity of the body, its contours, will be fully material, but materiality will be rethought as the effect of power, as power's most productive effect.”

More deeply conceived and as a recent step moving closer toward the conceptual depth underlying the notions of (re/dis)embodiment and version of ontological (in)security put forward in this article, Krickel-Choi (2021, 2) has emphasized that “ontological security-seeking actors are embodied and that this fact matters for their sense of being and their subsequent actions.” However, Krickel-Choi, like Narozhna, takes a relational approach by intervening to demonstrate “the interrelation of physical and ontological security” (Krickel-Choi 2021). Krickel-Choi does this by returning to the texts of Giddens (1991) and Laing (1990) to demonstrate these “original” OST scholars’ appreciation of the sociological concept of embodiment before scaling this up to discuss the ontological security of the “state-body.” Here, using the case of the Japanese-North Korean “abduction issue” Krickel-Choi demonstrate the simultaneousness of physical and identity-based security and that “states, too, can be seen as having bodies and that the body of a sovereign state consists at a minimum of territory and citizens” (Krickel-Choi 2021, 13). This single article comes closest to engaging at the embodied and (re/dis)embodying depth of (in)security I am concerned.

Building on the kind of OSS taking its referent object(s) as collectives and particularly Krickel-Choi's recent intervention reminding us about OST's original embodiment through my lens, bodies politic (what Krickel-Choi calls “state-bodies”) should be considered as no less “real” than the embodied subject and therefore fall under the scope and purview of the theory of ontological (in)security put forward in this article. Moreover, as I have argued elsewhere (Purnell 2020, 2021), while *the body politic* as metaphor is a rhetorical device used to make political communities knowable and intelligible as particular kinds of human beings, it is due to the performative power of metaphor that bodies politic (like individual embodied subjects) materialize. Bodies politic too should, therefore, be understood as the performative effect of their interpellations, as subject to (re/dis)embodiment, and therefore as also liable to having their ontologically security threatened. Indeed, explaining why and explaining why George Lakoff (1991, 1) provocatively argued that “metaphors can kill,” bodies politic—just like people—are the result of an embodiment in the sense of a making-into-a-body and are composed of lively collective body parts—"a hybrid forum composed of nested sets of complex permeable bodies” as Stefanie Fishel (2017, 43) has eloquently described them.

As explained so far, the above-cited moves made in OSS have paved the way for and indeed led to my intervention, which in response to both state- and individual-“level” approaches brings an interdisciplinary and embodied lens to the paradigm. However, it is important to highlight that I by no means am intending to “improve” on state- or individual-focused variants of OST, which have great application for grappling with questions of identity, anxiety, belonging, and violence in our international system as the works cited demonstrate. Rather, my intervention offers up an alternative. Indeed, following Anthony Burke (2007, 12) to understand ontology as “a statement about the nature and identity of being,” my intervention is aimed both wider and deeper than state- and individual-based OST through my focus on the *process* of (re/dis)embodiment, which too is concerned with the nature of bodily being and more precisely with how bodies get and “keep” or lose their being *as* individual(ized) or collective(ized) bodies*.* Therefore and moreover, the theory of ontological (in)security put forward in this article takes no agent-centric *object* as its unit of analysis at all. Rather, the notion of ontological (in)security put forward in this article takes a new materialist and indeed post-humanist approach to build on recent post and decolonial efforts made within OSS in IR to decenter the individual embodied subject (see DeLeon 2020; Untalan 2020) by redirecting the focus of analysis toward pre-, intersubjective, and collective processes and practices through which individual and collective bodies come to be, not be, or become other. Indeed, the notion of ontological (in)security put forward here *may* capture and duly scrutinize the embodied subject and collective political units. However, these actors are understood as occupying only particular points along what Diana Coole (2005) describes as the non-anthropocentric “agentic spectrum,” which runs from the prepersonal and intersubjective to collective and in this way my intervention brings the development of OSS full circle and beyond by scaling back and further down the scaled-up sociological/psychoanalytical OSTs devised within OSS in order to deal with the discipline's traditional “main actor” of states and secondary focus on the very particular, yet problematically disembodied, individual.

In further emphasizing the politics and insecurity of bodies at this *deep* ontological level and explaining how the process of (re/dis)embodiment constantly (re)materializes bodies, this article builds on Filip Ejdus’ (2017, 3) intervention to OSS which underlines that “material environments . . . provide an important source of ontological security.” Indeed, dealing with the ontologically insecure materiality of embodied subjects throughout, bodies are understood as coming to form part of the material environments, which for Ejdus contribute toward the ontological security of other actors while foregrounding the implications of such deep and profound ontological insecurity for the embodied subject itself. Finally, as a contribution to OSS wide enough to allow for the consideration of the (re/dis)embodiment of individuals and collective bodies and underlining how individuals and collective bodies correspond and indeed materialize as collective body parts and bodies politic, this article takes forward Brent Steele's (2013) conception of the scar by considering how individuals exist and function as parts of collectives, therefore not only leaving scars in their absence but also causing malaise and contributing toward the (re/dis)embodiment of collectives through their use and abuse in life and death. Indeed, as I envisage it, bodies politic become intelligible with a series of properties characteristic of particular embodied subjects—with organs and limbs if we take the *head of state*, the arm in *arm*y, and *the public eye* as obvious examples. However, as I have argued elsewhere and demonstrate in this article, the bodies politic coming to populate the contemporary international system are considerable outdated—based on enlightenment era, dualistic knowledge about bodies making *the body politic* an unwell rather than dead metaphor and life for such bodies extremely macho, violence prone, and unnecessarily nasty, brutish, and short (see Purnell 2021).

### Excessive Bodies, Vulnerable Bodies, and Ontological (In)Security

Bodily excess and vulnerability lead to the ontological depth of (in)security outlined in this article, and therefore both tenants require further expansion here. Indeed, materially and biologically, embodied subjects constantly exceed themselves by intentionally and sometimes unintentionally overspilling and leaking (as individual, bounded, Cartesian bodies enslaved by the mind should not). Thus, while some human bodies come to have legal and political rights, no amount of security, police, or law enforcement can prevent blood, sweat, and tears from seeping out of any body. However, on top of physically overspilling themselves, bodies are existentially and ontologically excessive—to the point that they can literally not be without others. This makes bodies very vulnerable indeed. In this section, I further expand on the notions of ontological and existential excesses and outline how these contribute toward processes of (re/dis)embodiment.

Coming to be via the intersubjective (and therefore excessive), social–political process of (re/dis)embodiment, embodied subjects are literally nobody—not a body—without each other. Thus, Butler (2015, 97) has emphasized how “even as located beings, we are always elsewhere, constituted in a sociality that exceeds us” while within IR, Edkins (2011, viii) agrees that “who people are is very bound up with who they are in relation to others.” Always and already insecure at the depth of being in itself and ontologically and existentially excessive due to reliance on outsiders knowing us *as embodied* to *be* at all, embodied subjects are inherently vulnerable. Following Brown, Jason, and Dorothy (2021, 4) to understand vulnerability as “a fundamentally ambivalent and ambiguous aspect of embodied, intercorporeal existence,” which is “constitutive and potentially generative component of embodied life” (Brown, Jason, and Dorothy 2021), vulnerability is therefore understood as contributing toward the depth of ontological insecurity outlined in this article while the process of (re/dis)embodiment I have described demonstrates just how such vulnerability implies a constitutive and/or destructive (and therefore “ambivalent”) unending process of bodily making, remaking, and unmaking.

Due to such existential and ontological excess, and the deep vulnerability (and therefore ontological insecurity) of the embodied subject, the ontological (in)security described in this article reflects vulnerability at the level of bodily being in itself. However, the extent of vulnerability in turn depends on the continually socially–politically contested process of (re)embodiment and in short means that while some bodies are made more vulnerable than others, some have their ontological security protected. Indeed, such protection or precariousness gradually accumulates via the repetition of speech acts and practices, which come to make bodies more or less secure. Moreover and crucially, *any body* can potentially and again gradually become known, used, and therefore *be* something else. Nicole Wegner's recent (Wegner 2021, 3) exploration of “the rituals and rhythms of war commemorations” certainly picks up on the ability of embodied subjects to be *made* as such in the eyes of others with Wegner underlining “the ways that these events recreate ethico-political relationships and subjectivities among participants.” Indeed, I too (see Purnell 2018) have previously discussed the contest of soldier's bodies by opposing actors viewing, valuing, and indeed knowing them differently—as a generic yet “precious resource” to fuel an unending war on the one hand and as unique persons on the other hand. However, in the following section, I go beyond the case of militarism and the contested bodies of the war dead to describe the raced, classed, and gendered, local–national–global lines along which every body is hierarchically arranged and (in)secured within the context of the COVID-19 pandemic.

Within existing, individual-focused OSS, there is some recognition of the implications of excess, with Greg Noble (2005, 107) explaining ontological security as “built on mutual recognition” and “fundamental to our capacity for social agency” going on to argue that “experiences of racism, especially since 2001, however, undermine the ability of migrants to feel ‘at home’, and hence their capacity to exist as citizens.” Toward illustrating his claim, Noble uses this article to “explore the ways in which migrants are increasingly made uncomfortable in Australia through acts of social incivility, harassment and abuse” (Noble 2005, 111), using examples of school bullying and public taunting of migrants in Australia after 9/11. However, and as before, my argument goes deeper due to taking the process of (re/dis)embodiment into account and meaning that on top of the possibility of becoming unable to “act socially” and “exist as citizens,” ontological (in)security also contains the possibility that failure to be *seen* and *regarded* as an embodied subject by others leaves one liable to becoming disembodied in a literal sense. In this way, I rather follow Butler by seeing the “interdependence of our embodied social life” (Butler 2020, par. 5) and, therefore, to regard the social and embodied as con-constituted and constitutive.

What is at stake given the existential depth of the ontological (in)security I have described this far is literal disembodiment, wherein those previously known and appearing as embodied subjects cease to be as such. Such examples are more commonly found within a context of violent warfare and I have written about these elsewhere (see Purnell 2021). Indeed, the cases of suicide bombing, disfigurement by torture, and the desecration of enemy corpses demonstrate the materialization of exclusionary and violent political discourse and are most easily found within the context of warfare. In everyday life, the process of (re/dis)embodiment—keeping us together and making us recognizable to one other as somebodies—is much more subtle but no less political. Indeed, the stakes are just as high. The following section, therefore, introduces the necropolitical context within which all contemporary (re/dis)embodiments take place and out of which the COVID-19 pandemic has emerged to threaten every body in often more subtle but no less deadly consequences for those made ontologically insecure and disembodied.

### Necropolitics and the (Ab)Use of Bodies

Necropolitics is a mode of governance oriented around death and concentrated on the production and division of populations into (1) those allowed, encouraged, and even made to live and (2) populations of others allowed and even required to die. Indeed, *necropolitics* is a term coined by Achille Mbembe (2003, 39) to better capture “contemporary forms of subjugation of life to the power of death” and finding biopolitical paradigm “insufficient” (Mbembe 2003) for the purpose. Crucially, as spatialized Mbembe highlights key features of necropolitical landscapes as “death-worlds” (Mbembe 2003, 40) within which certain people are reduced to the status of *the* “living dead” (Mbembe 2003). Moreover, Mbembe's necropolitical theory provides the case of European colonial practice of slavery as the paradigmatic example through which to showcase the dreadful potential of the necropolitical mode of governance underlining that “it would be a mistake to believe that we have left behind the regime that began with the slave trade and flourished in plantation and extraction colonies” (Mbembe 2013, 13). However, providing an extreme example of necropolitical control due to being “kept alive but in a state of injury, in a phantom-like world of horrors and intense cruelty and profanity” (Mbembe 2003, 21), the figure of the slave also provides an extreme example of ontological *in*security leading to disembodiment as the slave is reduced beyond the status of (body)part to become nothing more than fuel to be expended in the service of others and, of course, in the service of profit. As Mbembe (14/12/2018: par. 13) explains:

This living flesh has an economic value that can be, as suits the occasion, measured and quantified. A price can be attached to it. The matter produced from the brow sweat of slaves also has an active value insofar as the slave transforms nature, converts energy into matter, is itself at once a material, an energy-giving figure.

Necropolitical theory predating the pandemic well explains the role of race in dividing populations into (a) those allowed, encouraged, and even made to live and (b) those allowed and even required to die. During the COVID-19 pandemic, this transpired on both sides of the Atlantic as racialized populations came to comprise a disproportionate amount of both country's death tolls (see, e.g., Laurencin and Walker 2020; Cheng and Conca-Cheng 2020; Bhatia 2020; Khunti et al. 2020; Meer et al. 2020; Public Health England 2020). However, in his *Critique of Black Reason* (2013), Mbembe demonstrates how the principle of race has been used to gradually extend the category of expendable bodies, deemed suitable for being exposed to the power of death and used up in service, outward to encompass not only the slave but also all black men before including all of subaltern humanity and, finally, engulfing everybody.

Most recently and again writing on the necropolitics of the COVID-19 pandemic itself, Mbembe (2020a, par. 7) has noted the intersectional complexity of the present-day dividing practices acknowledging that while “in theory, the coronavirus can kill everyone,” some are able to “escape or delay death” (Mbembe 2020a) reminding us that:

This system always functioned on the basis of an apparatus of calculation; the idea that some are worth more than others.  And who is without value can be discarded.  The question is what we do with those whom we decide to be without value.  This question of course always affects the same races, the same social classes, the same genders.

Race is not the only factor determining the use, abuse, and relative ontological (in)security in a time of pandemic. However, toward more precisely articulating the nature of the shift engendering the increasing prevalence of necropolitics, in the time of coronavirus, Mbembe (2020a, par. 7) has gone as far as to conflate neoliberalism with necropolitics—speaking during the pandemic of “neoliberalism which we should call necroliberalism.” Speaking to the necroliberal conflation, on May 26, 2020, as US deaths from COVID-19 pushed 100,000, a White House Economic Advisor, Kevin Hassett, appeared live on CNN to declare that “our human capital stock is ready to go back to work” (cited in Naughtie 2020, par. 1). Causing immediate outcry, this deeply impersonal and dehumanizing phrase went “viral” with those offended by the term highlighting its connotations with slavery and white America's history of keeping and counting particular humans as chattel. However, in other circles, Hassett was defended by, for example, Slate's Jordan Weissman (2020) describing what happened as a technocratic slip up saying, “econo-speak made him sound a bit like a callous dork” and economist Gray Kimbrough (2020) tweeting “totally normal way for an economist to refer to people” as a caption to the CNN clip. Indeed, as totally normalized, the reduction of humans to “stock” speaks volumes to the endangerment and use of workers throughout the pandemic while demonstrating the necroliberal expansion of bodily dividing practices and ontological security threat outward from the category of slave.

As another example through which to demonstrate the depth of ontological insecurity and process of (re/dis)embodiment as well as the logics by which some bodies become other in a time of pandemic are observations made by the US President Donald Trump during the pandemic's first wave in 2020. Here, Trump (2020) describes watching freezer trucks arriving at the Elmhurst Hospital in New York reporting on March 29 in a televised White House address that:

they're pulling up to take out bodies, and you look inside and you see the black body bags. You say, ‘What's in there?’ It's Elmhurst Hospital; must be supplies. It's not supplies. It's people. I've never seen anything like it.

In this seeming digression from his scripted COVID-19 pandemic briefing (which was at this time a daily occurrence), the President crucially tells of his initial failure to realize that he was watching human body disposal on television, having mistakenly assumed that the black body bags were carrying medical “supplies.” From my perspective, his mistake is understandable but also telling of the process of disembodiment, which makes plain the deep ontological insecurity shared by every body and meaning that particular bodies may become unknowable as human and are treated accordingly. Indeed, in life, the bodies in those black bags may highly valued—by both their loved ones and more generally—for their functions within necrocapitalism. However, upon their expiry, they have become seemingly worthless and are being disposed of accordingly. Indeed, as I contend throughout this article, becoming known and treated as other than human and in this case as bagged trash is an outcome of the process of (re/dis)embodiment enabled and facilitated by the precariousness of embodied subjects and discursive-emotional strategies of exclusion.

Toward further unraveling the contemporary (necro)politics, Ahmed's *Wilful Subjects* (Ahmed 2014), which precedes *What's the Use?* (Ahmed 2019), is in fact littered with bodies that become known as body parts and used and used up according to their functions. This text allows for further insight into the logics the determining relative ontological (in)security of particular populations, which bodies tend to get reduced to, used, and used up as (body) parts and therefore become disembodied in the service of other bodies. Indeed, in *Wilful Subjects*, Ahmed returns to the relation of individual bodies to collective bodies through the notion of “the social body” (Ahmed 2014, 100–1), showing how some embodied subjects can be (re)made as body parts “cut off from bodies” (Ahmed 2014, 108) or as the body parts of other bodies. In illustrating her claim, Ahmed provides examples of people reduced to parts due to their deviance or perceived uselessness by referring to “odd parts,” singled out for sticking out like a “sore thumb” or being “mouthy” and accordingly “reduced to the speaking part as being reduced to the wrong part” (Ahmed 2014, 154).

Asking then “who become the arms?” the explicitly gendered examples of the handmaiden, footman, as well as the generic factory worker demonstrate that the kinds of bodies reduced to these particular body parts have a particular (body) part to play and belong to the lower or “service class” (Ahmed 2014, 111–12). However, in addition to being classed and gendered, the question of who becomes the arm and therefore who will be disembodied toward being used up in the service of another body is of course, like the dividing practices discussed so far, also profoundly racialized for Ahmed. Indeed, toward outlining how bodies are selected for particular uses and accordingly used up, Ahmed discusses the European colonial project at length, describing it as a project “justified as using what is unused” (Ahmed 2019, 47). Indeed, Ahmed's reading emphasizes how the European colonizer's narrative of discovering “unspoilt” lands and “lazy” and “idle” natives facilitated the putting to work by force and unto death as parts within the colonial machine in the case of slavery. Moreover, and again unfitting with the tendency of death makers to mask themselves with a layer of biopolitical rhetoric, Ahmed explains how in the case of European colonialism, “the extraction of use from bodies by defining others as bodies to be used was justified as a mission to improve humanity: to become an arm is to become body, to offer brawn and brute strength; to become arm as to become biological” (Ahmed 2019, 95). However, beyond race, Ahmed highlights the role of class in determining the bodies that will be reduced to parts and used accordingly. However, Ahmed does this through emphasizing the use of industrialising Britain's factory workers as parts within the capitalist machine. In particular, Ahmed does this by citing the frequency of factory floor accidents and lost limbs and how the factory workers retained their value and *use* as a laborer only as long as their hands remained “handy” (Ahmed 2014, 109) and after which as *used up*, parts become waste; therefore, Michelle Yeats (2011, 1679) has argued that “the body of the laborer is used up or wasted at accelerated rates so as to secure the most profit.”

As raced, gendered, and classed, the experience of being used and used up is extractive and exploitative and in this way works to make profit while materially (re)embodying those profiting too. This occurs as the exploitative reduction and use of another provide the exploitative user with “freedom from function” (Ahmed 2014, 112), enabling increased health, prosperity, and rest time and in turn materially (re)embodying those extracting service from and using others as parts. As Ahmed describes it, “some bodies will become arms, some bodies will employ others as arms” (Ahmed 2014, 108). Indeed, to draw on my own experience, during the COVID-19 pandemic, parts lower down the endoskeleton of the world were being used up, while other bodies, in circles closer to mine, were initially freed up from commutes and were able to work from home by using their newfound leisure time to exercise and bake. I found myself freed up to switch my empirical focus to the pandemic itself and went on to write my first book on it (see Purnell 2021). Through this time, I was serviced and shielded by others who, for example, I paid to deliver food to my door and take on the risk of COVID-19 exposure for me. However, at such a time of sped-up and exaggerated necropolitical exposures, as the body of the user is freed up and preserved, the material extraction involved in being used wears the part down and away ever more rapidly too. In this way, the parts being used and used up through their labor in both the time of COVID-19 and the time prior more slowly and subtly have a secondary function as coming to literally stand in for the part of the other and playing a role in the others’ at least temporary emancipatory relief from service and in this way literally buying them time unto death, which of course comes to all in the end and comes increasingly quickly in a time of pandemic. However, what is becoming clear through raced, classed, and gendered pandemic death tolls is that ontological (in)security is a zero-sum game as the preservation of some body entails the use of another. Indeed, within the context of the pandemic, COVID-19 is not equally threatening to every body and as scholars of IR we know this, as critical security studies tell us that both security and threat are socially–politically constructed. In the case of COVID, the virus is *made more* threatening as certain bodies are made less secure—physically and at the ontological levels of both identity and material being as bodies—as they are reduced discursively and then literally to parts in this case to be used and used up in the service of others.

As a final example, illustrating the misuse of bodies and as parts in the service of increasingly unhealthy bodies politic, the UK government's policy responses to COVID-19 further reveal how the persistence of outdated bodily metaphors used within the general public and policy circles to (mis)explain threats posed to human bodies materialize. These bodies politic materialize as apparently strong and independent bodies, but the pandemic has revealed them to be inherently vulnerable and precarious—unable and ill equipped to cope with COVID-19 and accordingly facing economic, social, and political crises of an entirely new order. For example, the ontological security of the UK body politic could be seen shifting with the onset of the COVID-19 pandemic, as Johnson (cited in Krishna 2020) suggested in March 2020 that the United Kingdom should “take it [COVID-19] on the chin” and sparked my investigation into the composition of the United Kingdom's raced, classed, gendered, and devalued “chin part” comprised disproportionately of those constructed as disposable and without a use according to the necropolitical logic outlined above: the elderly and already infirm and therefore unable to work as a part in the service of other bodies. Moreover, as the pandemic went on, the United Kingdom's National Health Service (NHS) was rhetorically transformed into “the beating heart” (cited in Stubley 2020, par. 1) of the UK body politic by Prime Minister Johnson while remaining on the verge of being “overwhelmed” throughout 2020 and 2021 and as the NHS staff were disproportionately used up in their service. However, with embodied subjects’ ontological (in)security and (re/dis)embodiments engendered exactly by political preferences, a rhetorical and political—more humane—reorientation might have seen such vulnerable bodies themselves placed at the center and becoming the objects of Johnson's rhetoric and concern and the NHS (re)constructed as a provider of their security, as a decent employer ensuring safe working conditions in a time of national crisis rather than the population being instructed and most vulnerable being let die in order to “protect the NHS” and “keep life moving” as the United Kingdom's national pandemic mottos instructed.

### What is the Use of Bodies within OSS?

As a process to which every body is subject, in this article, I have demonstrated how taking (re/dis)embodiment into account within the analysis of global politics entails widening and deepening OSS to capture and scrutinize the sometimes subtle but always ongoing contest of both individual(ized) and collective(ized) bodies, which arises from their existential precariousness as contested sites of local–global politics. Toward unravelling the contemporary politics of (re/dis)embodiment and determining the bodies more/less liable to becoming severely ontologically insecure *as bodies*, I have also described, intersectional racialized, gendered, and classed dividing practices’ work to disembody humans and put them to work as functional parts in the service of other but no less *embodied* and ontologically insecure collectives. However, as a primarily theoretical contribution, it is now my hope that scholars will take on the task of writing about patterns of (re/dis)embodiment and ontological (in)security in diverse empirical settings.

Written in and about this time of pandemic, in this article, I have presented this “wider” and “deeper” concept of ontological (in)security with an antifoundational epistemological approach and ontology and drawing and building on Mbembe's theses on *Necropolitics* (Mbembe 2003, 2019) and Ahmed's understanding of the relation between body parts and collective bodies (as put forward in *Wilful Subjects* [2014] and *What's The Use?* [2019]) to demonstrate how those known to themselves and others as human persons, as *somebodies*, can simultaneously become seen, used, and *be* less and other than bodies. I have also shown how such reduced and othered bodies can be reduced to and come to count as less than human while their value simultaneously increases as a body part. In this way, I have demonstrated the stakes of (re/dis)embodiment and the ontological insecurity it engenders by explaining how contemporary necropolitics involves bodies that are valued highly in life due to the function of the parts to which they have been reduced becoming instantly disposable on death, treated as waste, and, unless others demand differently, dumped as trash. Having served their purpose and other bodies in life, in the end, wasted parts are treated as such—excreted as a form of waste. Moreover, I have contextualized these dynamics of what has been described as (re/dis)embodiment through cases drawn from the COVID-19 pandemic by describing how particular bodies deemed useful for their function became reduced to parts, used, and used up in the service other—collective(ized) but no less *embodied bodies* including the NHS and body politic—for the purpose of maintaining a local–global necropolitical order based itself on a model of racist, classist, sexist, ageist, and albeist bodily extraction. In sum, I have contributed to OSS and widened and deepened the notion of ontological security in two ways: (1) OSS literature is primarily concerned with the ontological security of either states or individuals while I have widened this out to enquire into the ontological (in)security of what come to be known as and thus come to be bodies through the ongoing process of (re)embodiment and (2) by taking the ontological security of bodies as the primary focus, the notion put forward in this article also moved beyond dualist thinking such as that so often unthinkingly fallen back into within OSS prior to my intervention due to its concerned with “security not of the body, but of the self” (Mitzen 2006a, 344).

## Supplementary Material

ksab037_Supplemental_File
