# Corrigendum To: Bodies Coming Apart and Bodies Becoming Parts: Widening, Deepening, and Embodying Ontological (In) Security in the Context of the COVID-19 Pandemic

**DOI:** 10.1093/isagsq/ksab047

**Published:** 2021-12-17

Global Studies Quarterly (2021), https://doi.org/10.1093/isagsq/ksab037

In the originally published version of this manuscript, there was an error in a date. The begining of the first sentence of the Introduction: “In April 2021,” instead of “On May 25, 2020,”. This error is now corrected.

