# Supplementary material for: Bodies Coming Apart and Bodies Becoming Parts: Widening, Deepening, and Embodying Ontological (In)Security in the Context of the COVID-19 Pandemic
Source: Global Studies Quarterly. 2021 Nov 9;1(4):ksab037. doi: 10.1093/isagsq/ksab037 (PMC8689955; doi:10.1093/isagsq/ksab037)
Supplement: ksab037_Supplemental_File [file ksab037_supplemental_file.docx]

Author Biography

[Dr Kandida Purnell](https://kandidapurnell.me/) is an Assistant Professor of International Relations (IR) at Richmond, The American International University in London with a decade of experience in the English, US, and Scottish systems. Kandida’s research focuses on local-global body politics and takes an interdisciplinary, methodologically innovative, feminist, and de-colonial approach that draws and builds on contemporary international social and political theory by investigating dynamics of violence, embodiment, and society. Kandida’s work has explored cases including the covid-19 pandemic, the Global War on Terror (GWoT), mass casualty events, war repatriations, (un)commemorations, performances, and resistance practices. Kandida’s monograph ‘[Rethinking the Body in Global Politics’](https://www.routledge.com/Rethinking-the-Body-in-Global-Politics-Bodies-Body-Politics-and-the-Body/Purnell/p/book/9781138337329) was published by Routledge in 2021.

Acknowledgements

The theoretical-conceptual interventions made in this article stems from the doctoral research that I undertook under the supervision of Marysia Zalewski, Andrea Teti as well as the book project which developed to consider the body politics of the COVID-19 pandemic under the editorial guidance of Jenny Edkins and Nick Vaughn-Williams. However, as this article developed, the GSQ reviewers and editors provided recommendations which saw this my work developing substantially to further engage Ontological Security Studies in IR and for this I am extremely grateful.
